# Recombinant versus highly-purified, urinary follicle-stimulating hormone (r-FSH vs. HP-uFSH) in ovulation induction: a prospective, randomized study with cost-minimization analysis

**DOI:** 10.1186/1477-7827-4-38

**Published:** 2006-07-18

**Authors:** Alberto Revelli, Francesca Poso, Gianluca Gennarelli, Federica Moffa, Giuseppina Grassi, Marco Massobrio

**Affiliations:** 1 Reproductive Medicine and IVF Unit, Department of Obstetrical and Gynecological Sciences, University of Torino, S. Anna Hospital, Torino, Italy

## Abstract

**Background:**

Both recombinant FSH (r-FSH) and highly-purified, urinary FSH (HP-uFSH) are frequently used in ovulation induction associated with timed sexual intercourse. Their effectiveness is reported to be similar, and therefore the costs of treatment represent a major issue to be considered. Although several studies about costs in IVF have been published, data obtained in low-technology infertility treatments are still scarce.

**Methods:**

Two hundred and sixty infertile women (184 with unexplained infertility, 76 with CC-resistant polycystic ovary syndrome) at their first treatment cycle were randomized and included in the study. Ovulation induction was accomplished by daily administration of rFSH or HP-uFSH according to a low-dose, step-up regimen aimed to obtain a monofollicular ovulation. A bi- or tri-follicular ovulation was anyway accepted, whereas hCG was withdrawn and the cycle cancelled when more than three follicles greater than or equal to 18 mm diameter were seen at ultrasound. The primary outcome measure was the cost of therapy per delivered baby, estimated according to a cost-minimization analysis. Secondary outcomes were the following: monofollicular ovulation rate, total FSH dose, cycle cancellation rate, length of the follicular phase, number of developing follicles (>12 mm diameter), endometrial thickness at hCG, incidence of twinning and ovarian hyperstimulation syndrome, delivery rate.

**Results:**

The overall FSH dose needed to achieve ovulation was significantly lower with r-FSH, whereas all the other studied variables did not significantly differ with either treatments. However, a trend toward a higher delivery rate with r-FSH was observed in the whole group and also when results were considered subgrouping patients according to the indication to treatment.

**Conclusion:**

Considering the significantly lower number of vials/patient and the slight (although non-significant) increase in the delivery rate with r-FSH, the cost-minimization analysis showed a 9.4% reduction in the overall therapy cost per born baby in favor of r-FSH.

## Introduction

In the last decades, follicle-stimulating hormone (FSH) has assumed a central role in ovulation induction and has been shown to be highly effective in achieving ovulation in anovulatory infertile woman as well as in ovulation induction protocols for subfertile, ovulatory women and in superovulation for IVF [1]. Various FSH-containing products, both derived from extraction and purification from urine or from recombinant in vitro technology, have been developed in these years.

Recombinant FSH (r-FSH) and urinary FSH (u-FSH) have been repeatedly compared in trials dealing with superovulation induction for IVF. In some of this studies, r-FSH was reported to yield a better ovarian response with a higher number of retrieved oocytes [2-5], and a significantly lower total FSH dose [2-4,6]. The overall number of embryos obtained in IVF [2-4,7] and the pregnancy rate, calculated per started cycle or per transferred embryo, were also reported to be higher with r-FSH than with HP-uFSH in some of these studies [3,8].

On the other hand, only a few trials have compared r-FSH and uFSH in subfertile patients undergoing ovulation induction associated with intrauterine insemination (IUI) or timed sexual intercourse [9-14]. The limits of these studies are mainly two: a) some of them compared r-FSH to uFSH (and not to the more pure formulation HP-uFSH) [11-15], and b. some authors have considered as a group anovulatory, infertile women belonging to WHO group II without indicating the proportion of clomiphene citrate (CC)-resistant PCOS patients within the group [13]. When metanalyzed together, the above mentioned clinical trials have not allowed to draw definite conclusions about the relative effectiveness of r-FSH and u-FSH (or HP-uFSH) in patients undergoing low-technology assisted reproduction therapies [16], indicating the need for further studies.

From the economical perspective, two studies calculated the cost-effectiveness of r-FSH and u-FSH treatment in infertile patients undergoing induction of ovulation associated with IUI, and concluded that the urinary preparation was more cost-effective [17,18]. Even to this respect, however, conclusive data have not been provided so far.

The present trial was designed to compare the cost of the therapy with either r-FSH or highly-purified, urinary FSH (HP-uFSH) in patients submitted to ovulation induction. Both normoovulatory women with unexplained infertility and women with CC-resistant polycystic ovary syndrome (PCOS) were randomized in this prospective study. Assuming that the effectiveness of the two drugs in terms of pregnancy rate is similar [11-18], a cost-minimization analysis considering the cost sustained by the Italian Health Service for every delivered baby after FSH therapy was accomplished.

## Materials and methods

### Patients

Two hundred and sixty women belonging to subfertile couples undergoing ovulation induction associated with timed intercourse at the Reproductive Medicine and IVF Unit of the University of Turin were enrolled in this study.

Their age ranged between 28 and 38 years (mean ± SD 32.7 ± 4.3) and their body mass index (BMI) ranged between 19 and 26 Kg/m^2 ^(mean ± SD 21.5 ± 3.4); all were in good physical and mental health with no history of alcohol and/or drug abuse within the 24 months preceding the treatment. These patients had a history of couple's infertility from at least one year (mean ± SD 2.6 ± 1.5 yrs), primary infertility in about 75% of cases, secundary in about 25%. At a routinary diagnostic workout, patent tubes and normal uterine cavity were documented in all patients by a recent (within 1 year) hysterosalpingography or laparoscopy. A recent (within 2 months) semen analysis of the partner revealed normal semen parameters according to the World Health Organization standards [19].

Both normoovulatory patients with unexplained infertility (n = 184) and CC-resistant PCOS (n = 76) were included in the study. In the latter patients, CC resistance was defined as consistent failure to ovulate with incremental dose of CC up to 200 mg/day for 5 days in three previous treatment cycles.

### Study design

This prospective, randomized study aimed to compare the cost of therapy with either r-FSH (Gonal-F^® ^Serono, Switzerland) or HP-uFSH (Metrodin HP^® ^Serono, Switzerland) in women undergoing ovulation induction associated with timed intercourse.

Randomization was performed using a computer-generated random assignment schedule for each patient and was accomplished using a blocking method that assured an equal number of patients in the two treatment groups as well as a similar proportion of PCOS patients and of secondary infertility in the two groups. Only the first ovulation induction cycle of each patient was considered in the study. Overall, a total of number of 130 patients per group were included.

The treatment cost per delivered baby was considered as the primary end-point and on this basis the size of the study population was calculated by power analysis. The sample size calculation was performed using Graph-Pad StateMate version 2.0 (GraphPad Software, Inc. USA) as follows. Assuming from evidence of previous studies [9-12,14,17,18] a total amount of -20% ampules needed to reach ovulation in favour of r-FSH, the costs fixed by the Italian Health Service for 100 ampules of HP-uFSH and 80 ampules of r-FSH were calculated. This resulted in -17% cost in favour of HP-uFSH, assuming the effectiveness of both preparations to be the same. With such a difference, 130 observations per arm would be needed to have a detection power of 95% accepting a significance level of 0.05.

The following clinical parameters were used to accomplish a cost-minimization analysis (for details see paragraph below), and were registered as secondary end-points: monofollicular response rate, total FSH dose, cycle cancellation rate, length of the follicular phase; number of follicles between 12 and 17 mm the day of hCG administration; diameter of the leading follicle and endometrial thickness the day of hCG administration, twinning rate, incidence of ovarian hyperstimulation syndrome and delivery rate.

The ovulation induction outcome was defined as monofollicular when only one follicle ≥ 18 mm without other follicles ≥ 12 mm diameter was observed the day of hCG administration. When a bi- or tri-follicular development was recorded (two or three follicles ≥ 18 mm without other follicles ≥ 12 mm diameter the day of hCG administration), the treatment was continued and hCG was administered, whereas the treatment cycle was cancelled when more then three follicles developed up to 18 mm diameter or, conversely, no follicle developed to more than 12 mm diameter within 28 days of FSH administration.

### Ovulation induction regimen

Ovulation induction was aimed to obtain a monofollicular development and was accomplished using the low dose, step-up protocol starting on day 3 of a spontaneous or progesterone-induced withdrawal bleeding by means of subcutaneous or intramuscular injections of 75 IU/day of either r-FSH or HP-uFSH. If no ovarian response was detected after two weeks, the daily dose was increased to 112.5 IU for one week, and then to 150 IU. The ovarian stimulation was stopped in case of no ovarian response after 28 days of FSH administration.

Ovulation induction was monitored by vaginal ultrasound every second-third day starting on day 7–9 of the cycle; in case of no ovarian response with daily dose increase, ultrasound evaluation was postponed by one week.

Follicle rupture was induced by intramuscular injection of 10,000 IU human chorionic gonadotropin (hCG; Profasi HP^®^, Serono, Switzerland) when the leading follicle reached 20 mm diameter. Free sexual intercourses were encouraged from the day of hCG administration onward only in cases when no more than three follicles bigger than 17 mm were observed; otherwise, hCG was not administered and protected sexual intercourse was recommended in order to avoid high-order multiple conception.

The luteal phase was supported in every case by vaginal progesterone (Esolut^® ^Angelini, Italy) at a daily dose of 200 mg for 12 days starting on day +2 from hCG administration.

Pregnancy was assessed by serum hCG assay and confirmed by vaginal ultrasound at six weeks of amenorrhea. All pregnancies that ended with the delivery of a viable newborn were considered in calculating the delivery rate.

### Cost-minimization analysis

The cost-minimization analysis was accomplished considering the costs of FSH to the Italian Health Service (IHS), as IHS sustains the cost of FSH for these treatments and it is delivered free of charge to patients.

The cost of each treatment was calculated considering the cost per vial (75 IU) at the time of the clinical trial, that was equal to 23.40 € and 15.55 € for r-FSH and HP-uFSH, respectively. The economic impact of the other health resources on IHS in this trial (e.g. ultrasound monitoring) was the same for both study groups, and thus was not considered.

The final outcome of the cost-minimization analysis was to compare the cost of FSH treatment per delivered baby. Cost-minimization analysis was preferred to cost-effectiveness analysis because it fits better to comparative studies in which the effectiveness of compared treatments is assumed to be similar, as previously reported in this case [11-18]. Sensitivity analysis was performed calculating the cost per delivery after having considered both the mean number of FSH vials consumed in each study group and the delivery rate for started cycle around the 95% confidence interval.

### Statistical analysis

The JMP software (version 4.0.4; SAS Corp., Cary, NC) was used to perform statistical analysis. The parameters calculated for either HP-uFSH and r-FSH-treated patients were compared using the two-tailed Student's *t *test for independent data and the χ^2 ^test, setting the significance level at p ≤ 0.05.

## Results

A total number of 260 patients were randomized and included in the study, 130 in the HP-uFSH group and 130 in the r-FSH group; the clinical and endocrine characteristics of the patients in the two study groups were homogeneous (Table 1).

**Table 1:** Main characteristics of patients receiving ovarian stimulation by highly purified urinary human follicle stimulating hormone (HP-uFSH) or recombinant human follicle stimulating hormone (rFSH) (* values are expressed as mean ± SD; ns = not significant)

	Overall	HP-uFSH	rFSH	p
N. of patients	260	130	130	
Age (years) *	32.7 ± 4.3	33.0 ± 3.6	32.3 ± 4.0	ns
BMI (kg/m^2^) *	21.5 ± 3.4	21.2 ± 3.0	21.3 ± 3.1	ns
Duration of infertility (years) *	2.6 ± 1.5	2.7 ± 1.4	2.5 ± 1.4	ns
Type of infertility (%):				
Primary	196	97 (74.6)	99 (76.1)	ns
Secondary	64	33 (25.4)	31 (23.9)	ns
Indication to treatment (%):				
unexplained infertility	184	91 (70.0)	93 (71.5)	ns
CC-resistant PCOS	76	39 (30.0)	37 (28.5)	ns
LH/FSH *	1.4 ± 0.6	1.3 ± 0.9	1.4 ± 0.9	ns
Testosterone (ng/mL) *	0.6 ± 0.8	0.6 ± 0.5	0.7 ± 0.1	ns
Prolactin (ng/mL) *	17.9 ± 4.1	17.7 ± 4.0	18.3 ± 3.8	ns

Overall, 260 first-attempt treatment cycles were considered, 130 with HP-uFSH and 130 with r-FSH. Fifty cycles (19.2% of all) were cancelled, whereas 210 were concluded with hCG administration, among which 178 (68.5%) were monofollicular and 32 (12.3%) were bi-/trifollicular (Table 2). Thirty pregnancies reaching delivery were obtained, with an overall delivery rate per started cycle of 11.5% and for ovulatory cycle of 13.6% (Table 2). All pregnancies were singleton.

**Table 2:** Overall outcome of stimulation using a "low dose step-up" protocol and comparison between HP-uFSH and r-FSH (* values are expressed as mean ± SD; ns = not significant)

	overall	HP-uFSH	rFSH	P
N. of patients	260	130	130	
N. of stimulation cycles	260	130	130	
Total FSH dose (IU) *	733 ± 387	844 ± 305	668 ± 276	.0003
Duration of follicular phase (days) *	12.3 ± 2.5	12.7 ± 2.6	11.7 ± 2.5	ns
No. of preovulatory follicles ≥ 18 mm at hCG *	1.8 ± 1.2	1.9 ± 1.1	1.8 ± 1.2	ns
No. of follicles 12–17 mm at hCG *	3.8 ± 1.6	3.7 ± 1.9	3.8 ± 1.6	ns
Size of the dominant follicle at hCG (mm) *	20.2 ± 1.1	20.1 ± 1.0	20.3 ± 0.9	ns
Endometrial thickness at hCG (mm) *	10.4 ± 1.5	10.8 ± 1.1	9.9 ± 1.8	ns
Monofollicular cycles (%)	178 (68.5)	87 (66.9)	91 (70.0)	ns
Bi-/trifollicular cycles (%)	32 (12.3)	16 (12.3)	16 (12.3)	ns
Cancelled cycles (%)	50 (19.2)	27 (20.8)	23 (17.7)	ns
Deliveries	30	13	17	
Delivery rate/started cycle (%)	11.5	10.0 (95%CI: 4.5–15.2)	13.1 (95%CI: 7.3–18.9)	ns
Delivery rate/ovulatory cycle (%)	13.6	12.0	15.2	ns

The only parameter that differed significantly between the two study groups was the total FSH dose, that was significantly lower using r-FSH (p < 0.0003; Table 2). The monofollicular ovulation rate was not significantly different in women treated with HP-uFSH and those receiving r-FSH (66.9% vs. 70.0%, respectively); the same was observed for cancellation rate (20.8% and 17.7%, respectively) and proportion of bi-/trifollicular responses (12.3% in both groups) (Table 2).

Results of normoovulatory patients and PCOS patients are shown separately in Tables 3 and 4. In both these subgroups, the only significant difference was the total FSH dose, lower when r-FSH was used (Tables 3 and 4). Although r-FSH showed a higher effectiveness in terms of delivery rates in both subgroups, it was not significantly different (Tables 3 and 4).

**Table 3:** Outcome of stimulation in normoovulatory patients with unexplained infertility and comparison between HP-uFSH and r-FSH (* values are expressed as mean ± SD; ns = not significant).

	Overall	HP-uFSH	rFSH	P
N. of patients	184	91	93	
N. of stimulation cycles	184	91	93	
Total FSH dose (IU) *	762 ± 505	837 ± 349	644 ± 199	0.005
Duration of follicular phase (days) *	12.0 ± 2.9	12.4 ± 2.5	11.6 ± 2.1	ns
No. of preovulatory follicles ≥ 18 mm at hCG *	1.2 ± 0.9	1.2 ± 0.8	1.1 ± 0.9	ns
No. of follicles 12–17 mm at hCG *	0.4 ± 0.3	0.4 ± 0.3	0.4 ± 0.3	ns
Size of the dominant follicle at hCG (mm) *	20.5 ± 1.9	20.6 ± 1.8	20.4 ± 1. 8	ns
Endometrial thickness at hCG (mm) *	9.5 ± 2.0	9.6 ± 1.9	9.5 ± 1.5	ns
Monofollicular cycles (%)	150 (81.5)	74 (81.3)	76 (81.7)	ns
Bi-/trifollicular cycles (%)	20 (10.9)	10 (11.0)	10 (10.8)	ns
Cancelled cycles (%)	14 (7.6)	7 (7.7)	7 (7.5)	ns
Deliveries	19	9	10	
Delivery rate/started cycle (%)	10.3	9.9	10.7	ns
Delivery rate/ovulatory cycle (%)	11.2	10.7	11.6	ns

**Table 4:** Outcome of stimulation in patients with clomiphene citrate-resistant polycystic ovary syndrome and comparison between HP-uFSH and r-FSH (* values are expressed as mean ± SD; ns = not significant)

	Overall	HP-uFSH	rFSH	P
N. of patients	76	39	37	
N. of stimulation cycles	76	39	37	
Total FSH dose (IU) *	648 ± 498	833 ± 307	546 ± 346	0.002
Duration of follicular phase (days) *	12.1 ± 3.9	11.9 ± 3.6	12.2 ± 3.7	ns
No. of preovulatory follicles ≥ 18 mm at hCG *	2.0 ± 0.9	2.0 ± 0.8	2.0 ± 0.9	ns
No. of follicles 12–17 mm at hCG *	4.2 ± 2.1	4.2 ± 2.0	4.1 ± 1.9	ns
Size of the dominant follicle at hCG (mm) *	20.8 ± 1.8	20.8 ± 1.6	20.9 ± 1. 7	ns
Endometrial thickness at hCG (mm) *	10.7 ± 2.0	10.9 ± 1.7	10.5 ± 1.9	ns
Monofollicular cycles (%)	28 (36.8)	13 (33.3)	15 (40.5)	ns
Bi/trifollicular cycles (%)	12 (15.8)	6 (15.4)	6 (16.2)	ns
Cancelled cycles (%)	36 (47.4)	20 (51.3)	16 (43.2)	ns
Deliveries	11	4	7	
Delivery rate/started cycle (%)	14.5	10.3	18.9	ns
Delivery rate/ovulatory cycle (%)	27.5	21.1	33.3	ns

Both HP-uFSH and r-FSH were very well tolerated by patients and only one case of local injection-site eriythema was observed after HP-uFSH administration. No systemic adverse effects were observed and no severe hyperstimulation syndrome (OHSS) occurred.

Results of cost-minimization analysis are shown in Table 5. Patients treated with r-FSH required a 21.2% lower number of vials (75 IU) than patients treated with HP-uFSH group. Considering the 50.5% higher cost per vial of r-FSH, even with a lower vial consumption/treatment the cost per cycle in the r-FSH group resulted 18.5% higher than in the HP-uFSH group (Table 5). Taking into account the slightly higher (although not significant) effectiveness of r-FSH in terms of delivered pregnancies, the total number of FSH vials per delivered baby resulted to be 39.8% lower for patients treated with r-FSH (Table 5), and the final treatment cost per delivered baby was 9.4% lower in the r-FSH group (Table 5). In the sensitivity analysis, the cost for delivered baby resulted to be lower with r-FSH also after correcting for the confidence interval of delivery rate, the cost reduction ranging from 2.7% to 21.9% with rFSH.

**Table 5:** Cost-minimization analysis and (last row) percentage variation of economical parameters in r-FSH vs. HP-uFSH treatment

	Mean FSH IU/cycle (95%CI)	Mean n. of vials/cycle (95%CI) (A)	Cost per vial (€) (B)	Mean cost/cycle (€) (C) = (A) × (B)	Total n. of vials (95%CI) (D) = (A) × 130	N. of deliveries (95%CI) (E)	Total n. of vials/delivery (F) = (D)/(E)	Cost per delivery (€) (B) × (F)
HP-uFSH	844 (741–947)	11.3 (9.9–12.6)	15.55	175.71	1,469 (1,287–1,638)	13 (6–20)	113 (214–82)	1,757.15
r-FSH	668 (575–761)	8.9 (7.7–10.1)	23.40	208.26	1,157 (1,001–1,313)	17 (9–25)	68 (111–53)	1,591.20
%	-20.8	-21.2	+50.5	+18.5	-21.2	+30.8	-39.8	-9.4

## Discussion

The technology used to obtain pharmacologically available human FSH has traditionally been extraction and purification from the urine of postmenopausal women. The purification process has been progressively improved, finally yielding a highly-purified urinary FSH (HP-uFSH) with less than 0.001 IU of LH per FSH ampoule and a low amount of proteinic contamination. The increasing spread of assisted reproduction techniques that has taken place all over the world in the last years has rapidly increased the need of bulk amounts of FSH for therapeutic use, and a recombinant technology to get theoretically unlimited amounts of recombinant human FSH (r-FSH) from cultured cells has been successfully developed [20].

In comparison to uFSH or HP-uFSH, r-FSH has an absolute purity (no LH content, no contamination by proteinic molecules), a higher batch-to-batch consistency, and no risk of transmission of infectious diseases [20]. However, r-FSH is much more expensive than urinary FSH on a per-unit basis, and its cost-effectiveness remains controversial, especially for patients undergoing ovulation induction and low-technology assisted reproduction treatments [21].

Although r-FSH has been claimed to achieve some advantage in case of IVF treatment (more oocytes, more embryos available for transfer, a higher pregnancy chance with a lower total FSH dose and a shorter follicular phase) [2,4,7,8,22,23], only a few studies have been designed to compare r-FSH and uFSH in low-technology treatments, when monofollicular ovulation is preferable. Moreover, in most of these studies uFSH and not HP-uFSH was used [11-15,17,18], as only two of them compared rFSH to HP-uFSH in low-technology ART [9,10]. Furthermore, WHO group II anovulatory women were considered by some authors as an homogeneous group, without calculating the proportion of CC-resistant PCOS patients within the studied population [13]. When available data were pooled together and metanalyzed, no conclusive results could be found, and the need for further studies comparing r-FSH and uFSH was underlined [16].

In the present study, an FSH low dose, step-up protocol was adopted in order to get preferably monofollicular ovulation (with a few cases having two/three preovulatory follicles); all patients having more than three preovulatory follicles were discouraged to conceive: this minimized the risk of multiple pregnancy and ovarian hyperstimulation syndrome even in high-risk patients (e.g. young CC-resistant PCOS patients). Overall, a high proportion of ovulatory cycles and a high rate of monofollicular ovulation was obtained with either FSH formulation, very frequently (about 90% of cases) in normoovulatory women with unexplained infertility, in about 50% of cases in patients with CC-resistant PCOS, which are known to have a well defined trend toward multifollicular recruitment. In contrast with some of the reports comparing r-FSH to uFSH [12,13], we did not observe any significant difference between r-FSH and HP-uFSH as far as the cycle cancellation rate and the monofollicular ovulation rate are concerned. Probably the higher purity of HP-uFSH with respect to the less purified uFSH, and the consequent lower interference produced by LH and other proteinic contaminants over the dominant follicle selection, explains the discrepancy between previous studies [12,13] and ours. Indeed, our results are similar to those obtained by Matorras et al., that compared HP-uFSH to r-FSH in normoovulatory women undergoing IUI [10].

Consistently to previous reports [9-12,14,17,18], the total FSH dose necessary to achieve ovulation was significantly lower using r-FSH. The reason for this can likely be found in the higher biological potency of r-FSH, in turn linked to the more basic spectrum of isoforms that gives more receptor binding affinity to the molecule, as well as to the lower proportion of degraded FSH forms in r-FSH [24]. An alternative possibility is that r-FSH could be more active in inducing the synthesis of intraovarian factors (e.g. Inhibin A) able to amplify the effects of FSH at the ovarian level [25].

Consistently with previously published reports [9,10,12,13,17,18] the results in terms of deliveries per started cycle and per ovulatory cycle were not significantly different with either r-FSH or HP-uFSH, although the delivery rate was slightly higher with the recombinant product. The cancellation of every cycle in which more than three preovulatory follicles were developed prevented our patients from experiencing twin pregnancies or ovarian hyperstimulation syndrome, that are unlikely when less than four follicles are developed.

Since both r-FSH and HP-uFSH allow to achieve comparable results in low-technology cycles, the cost of therapy is a major issue to be considered. In IVF, the overall cost per successful pregnancy resulted to be significantly lower with r-FSH, due to its higher effectiveness [26]. However, in low-technology treatments like ovulation induction plus timed intercourse, the pregnancy rate is not significantly higher using r-FSH, and therefore the final cost per delivered baby using HP-uFSH or r-FSH needs a careful evaluation. Two recent studies calculated the cost-effectiveness of treatments with r-FSH or u-FSH in infertile patients undergoing induction of ovulation associated with IUI, and concluded that the urinary preparation was more cost-effective [17,18]. However, the cost-minimization analysis fits better to analyze costs in comparative studies in which the treatment effectiveness of the studied drugs is very similar, as in this case [11-18].

In our prospective, randomized trial the total number of FSH vials per delivered baby was about 40% lower among patients treated with r-FSH. For this reason and for the slightly higher delivery rate in the r-FSH group, even considering the higher cost per vial of r-FSH, the final cost per delivery was estimated 9.4% lower with r-FSH. The economical advantage of r-FSH was confirmed even when the confidence interval of delivery rate was considered in a sensitivity analysis; in fact, even after correction, the cost reduction per delivered baby was still ranging from 2.7% to 21.9% in favor of r-FSH.

It must be remarked that differently from the previously published cost-effectiveness studies [17,18], in our study only first-treatment cycles were considered, Hp-uFSH (and not u-FSH) was used, mainly normal (and not all PCOS) patients were included, and the delivery rate (and not just the clinical pregnancy rate) was considered as the best clinical outcome indicator.

In conclusion, the present study shows that both HP-uFSH and r-FSH can be safely and effectively used to induce ovulation induction both in normoovulatory patients with unexplained infertility and in CC-resistant PCOS patients. The slightly higher effectiveness of r-FSH in terms of delivered babies seems to compensate for the higher cost per IU, leading to lower final economical costs per delivered baby.
